# Study on clinical and biological characteristics of ameloblastic carcinoma

**DOI:** 10.1186/s13023-020-01603-5

**Published:** 2020-11-11

**Authors:** Zhixing Niu, Ye Li, Wantao Chen, Junfang Zhao, Hongyu Zheng, Qing Deng, Zhian Zha, Hao Zhu, Qiang Sun, Lei Su

**Affiliations:** 1 Department of Stomatology, The First Affiliated Hospital of Zhengzhou University, No. 1, East Jian she Road, Zhengzhou, 450052 Henan Province China; 2 Academy of Medical Sciences, Zhengzhou University, No. 100 Science Avenue, Zhengzhou, 450000 Henan Province China; 3 Department of Pathology, The First Affiliated Hospital of Zhengzhou University, No. 1, East Jian she Road, Zhengzhou, 450052 Henan Province China; 4 Department of Oral and Maxillofacial-Head and Neck Oncology, Ninth People’s Hospital, Shanghai Jiao Tong University School of Medicine, 639 Zhizaoju Road, Shanghai, 200011 China; 5 Department of Pathology, The First Affiliated Hospital of Henan University of Science and Technology, No. 24 Jinghua Road, Jianxi District, Luoyang City, 471003 Henan Province China; 6 Department of Radiology, The First Affiliated Hospital of Zhengzhou University, No. 1, East Jian she Road, Zhengzhou, Henan Province China

**Keywords:** Ameloblastic carcinoma, Ameloblastoma, *BRAF* gene, Targeted therapy

## Abstract

**Background:**

Ameloblastic carcinoma (AC) is an odontogenic malignant tumor which is closely related to benign ameloblastoma. Because of its rarity, diagnosis and treatment are difficult. In this study, we summarized and analyzed the clinical and biological characteristics of AC.

**Results:**

Fifteen patients with AC and a median age of 53 years were identified. Among of them, five patients who were tested carried a *BRAF-V600E* mutation. Two patients presented with cervical lymph nodes and lung metastases. Primary AC was more invasive, and the bone destruction ability of the primary type was more radical than that of the secondary type.

**Conclusions:**

This study revealed that the *BRAF-V600E* mutation was related to the aggressive behavior of AC, and early radical resection is crucial. Moreover, targeted therapy may be a new direction in the future.

## Background

Ameloblastoma is a common odontogenic epithelial tumor that can transform into a malignant tumor called ameloblastic carcinoma (AC), which is very rare [1]. In the latest edition of the 2017 World Health Organization (WHO) classification of odontogenic tumors, AC was defined as a rare odontogenic malignancy that combines the histologic features of ameloblastoma with cytologic atypia, having a 5-year survival rate of 69.1% [2, 3]. AC occurs mainly in the posterior mandible and presents as two main types: a primary type called de novo* cancer* and a secondary type, defined as a malignant transformation from a pre-existing benign ameloblastoma [3, 4].

Unfortunately, AC has a high recurrence rate after surgery, causing invasive and extensive bone destruction; its clinical diagnosis and treatment are very challenging. Transformation may be closely associated with a long medical history, multiple operations, radiotherapy, and chemotherapy, but the mechanisms of malignant transformation are poorly understood. Therefore, early tumor diagnosis and treatment are crucial.

In recent years, with the rapid development of molecular biology, some studies also reported a *BRAF-V600E* mutation rate of approximately 60% in ameloblastoma [5–7]. The *BRAF* gene is an important proto-oncogene that plays an important role in tumor cell proliferation, differentiation, and apoptosis. Therefore, the presence of *BRAF-V600E* mutation may be a biomarker of a more aggressive clinical course. Despite published reports on AC [2, 8], the systematic analysis of large samples of clinical, imaging, and pathological features is still lacking. In this study, we analyzed 15 patients with AC with a clear diagnosis and summarized the clinical and biological characteristics of AC.

## Results

### Clinical summary and manifestations

**Fig. 1:**
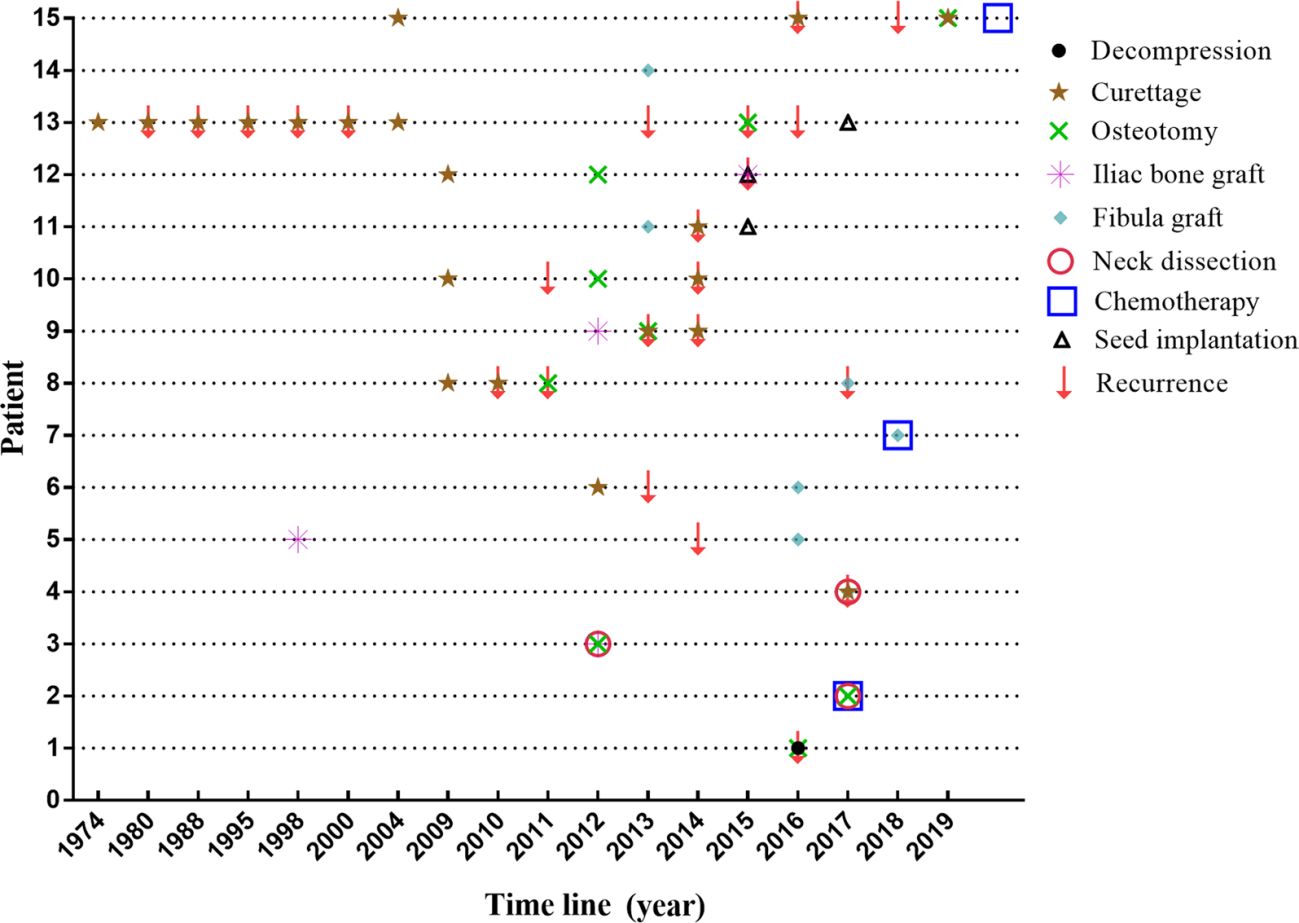
Time line

A total of 15 patients diagnosed with AC between 2014 and 2019 were included. The median age of the cohort was 53 (range 24–75) years. The duration of symptoms before diagnosis was 0.5–41 years, and the average disease duration was 10.3 years (Table 1). The mandible was found to be the most common tumor site in 86.7% of the patients (n = 13), followed by the maxilla (n = 2, 13.3%). Six patients had inferior alveolar nerve paralysis, and two had lymph node (Level Ib) and lung metastasis at presentation. Solid tumor/multicystic type structures were more common in 60% of the patients (n = 9), followed by the cystic (n = 4, 26.7%), and mixed type (n = 2, 13.3%). The patients’ clinical data are summarized in Tables 1 and 2. The varied treatment of these 15 AC cases included decompression, osteotomy, curettage, iliac bone graft, fibula graft, neck dissection, chemotherapy (Oxaliplatin, Tegafur), and seed implantation. So far, no patient has died. Conservative treatment such as curettage had a high recurrence rate, but radical resection and jaw reconstruction seemed to show satisfactory postoperative results (Table 1 and Fig. 1).

**Table 1:** Clinical information

Patient no	Structure	Diameter (cm)	Facial swelling	Tooth loosening	Limitation of mouth opening	Pain	Numbness	Growth direction
1	Cystic	4.9	Yes	No	No	+	Yes	Buccolingual
2	Solid	6.0	Yes	No	No	+++	Yes	Buccal
3	Solid	3.0	Yes	II°	Mild	+++	Yes	Buccal
4	Solid	8.0	No	No	Moderate	+++	No	Lingual
5	Solid	3.3	Yes	No	Mild	+	No	Submandibular
6	Cystic	2.0	Yes	III°	No	+++	No	Buccal
7	Mixed type	7.5	Yes	II°	No	+++	No	Buccal
8	Solid	2.5	No	No	No	-	No	Buccal
9	Cystic	4.0	Yes	No	No	+	Yes	Buccal
10	Cystic	8.5	Yes	II°	No	+++	Yes	Lingual
11	Solid	4.8	Yes	II°	No	+	No	Buccal
12	Solid	2.5	Yes	II°	No	++	No	Lingual
13	Mixed type	5.0	Yes	II°	No	-	No	Buccal
14	Solid	6.0	Yes	III°	No	+	Yes	Buccal
15	Cystic	8.0	Yes	No	No	+	No	Buccal

Mild(I°); moderate(II°); severe(III°)

**Table 2:** Clinical manifestation

Patient no	Location	Sex/year	S/C/R (times)	Course (year)	Follow-up time (month)	R-t/Treatment	BRAF-V600E	Type
1	Maxilla	F/53	2/0/0	1	32	1/Conservative	+	Primary
2	Mandible	M/64	1/3/0	0.5	26	0/Radical + ND + C	+	
3	Mandible	F/63	1/0/0	20	82	0/Radical + ND + RC	NP	
4	Mandible	M/52	2/0/0	0.5	21	1/Conservative	+	
5	Mandible	F/66	2/0/0	18	40	1/Radical + RC	NP	Secondary
6	Mandible	F/60	2/0/0	4.5	36	1/Conservative	NP	
7	Mandible	M/43	1/2/0	18	14	0/Radical + C	NP	
8	Mandible	F/28	4/0/0	8	21	2/Conservative; 1/Radical	NP	
9	Mandible	M/61	4/0/0	2.3	69	1/Radical + RC;1/Conservative; 1/Radical	+	
10	Mandible	F/75	3/0/0	5	78	1/Conservative; 1/Radical	NP	
11	Mandible	M/25	2/0/1	1.5	69	1/Radical + RC;1/Conservative	+	
12	Mandible	M/24	3/0/0	6	52	1/Conservative;1/Radical	NP	
13	Maxilla	F/68	8/0/1	41	54	6/Conservative;1/Radical	NP	
14	Mandible	M/51	1/0/0	13	67	0/Radical + RC	NP	
15	Mandible	M/36	4/1/0	15	5	3/Conservative	NP	

*F* Female; *M* Male; *S* Surgery; *C* Chemotherapy; *R* Radiotherapy; *R-t* Recurrence-times; *RC* Reconstruction; *ND* Neck dissection; *NP* Not performed

### Imaging features

**Fig. 2:**
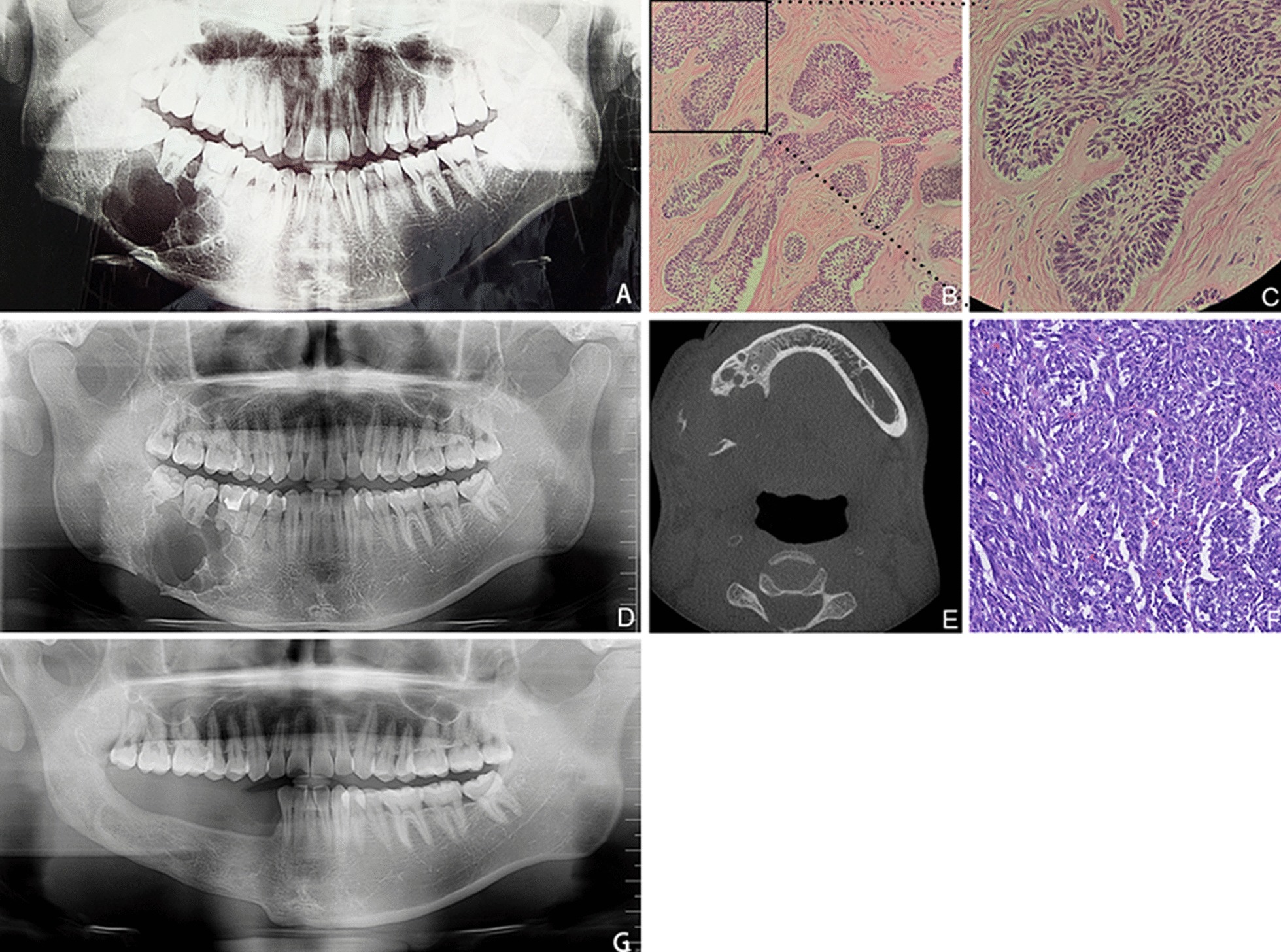
Patient 12, Secondary ameloblastic carcinoma: **a** A mass in the right mandible with irregular bone absorption and bone destruction. **b** Postoperative pathology revealed follicular ameloblastoma (HE, × 200). **c** The tumor epithelium shows columnar cells with palisade nuclei far from the basement membrane (HE, × 400). **d** Radiography revealed polycystic bone destruction in the left mandible and soap bubble-like and root truncation-like absorption after 3 postoperative days. **e** Cone beam computed tomography (CBCT) showed buccal-lingual bone destruction and polycystic tumors. **f** Postoperative pathology revealed ameloblastic carcinoma and cell atypia. **g** Radical excision and iliac bone transplantation were performed simultaneously, leading to satisfactory results 4 years postoperatively

AC manifests initially as multicystic or unicystic lesions with clear boundaries and a visible sclerotic zone. As the tumors increase in size, patients may experience tooth displacement, root resorption, osteolytic destruction, unclear boundaries, honeycomb-like changes (Fig. 2d), significantly uneven enhancement, further soft tissue invasion, infection, and facial swelling. Imaging revealed that primary tumors were more destructive than secondary tumors (Fig. 4).

### Pathological features

**Fig. 3:**
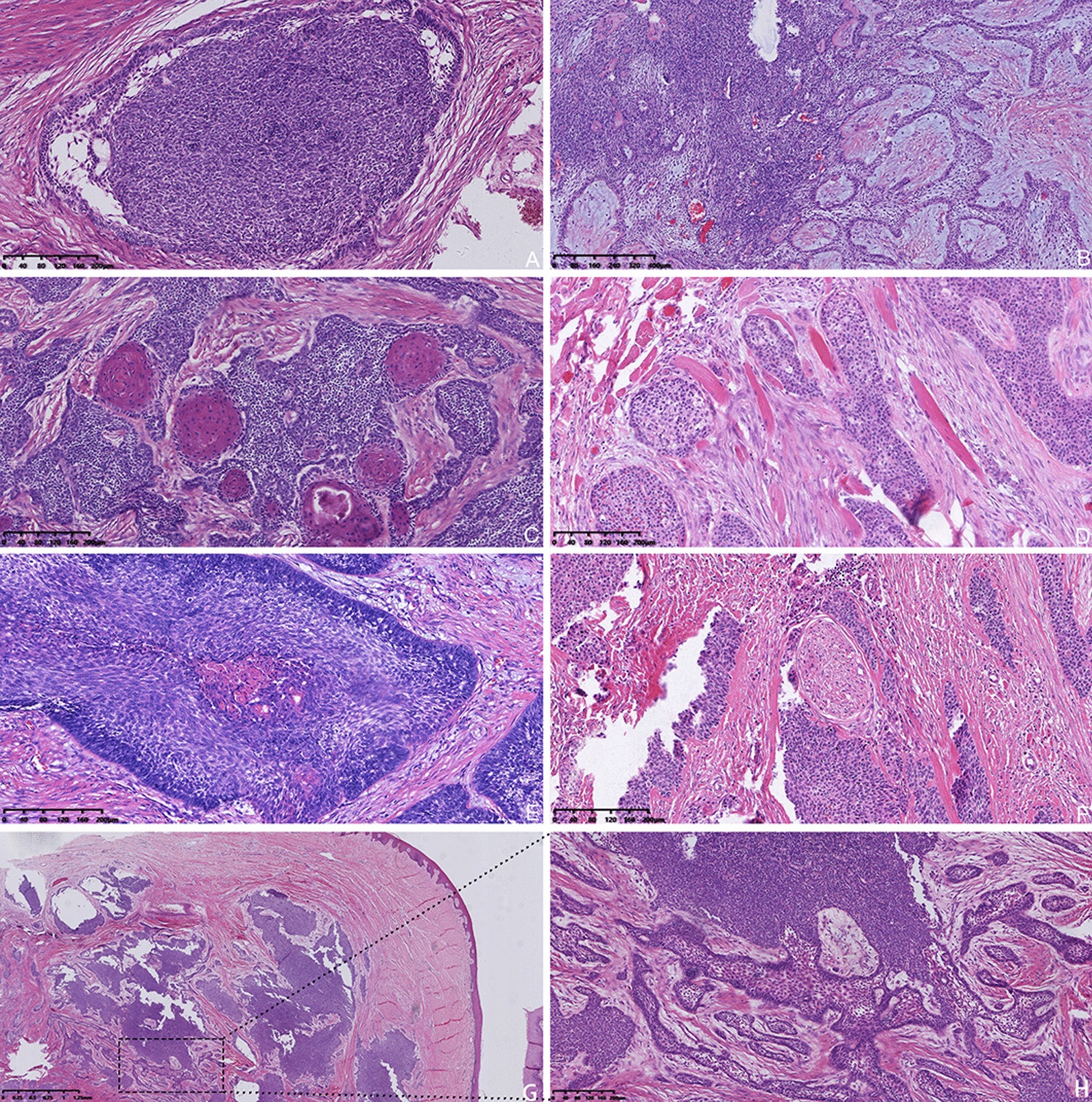
Hematoxylin and eosin (HE, × 100) staining of ameloblastic carcinoma. **a** Tumor epithelial dysplasia. **b** Canceration is observed at the junction of the tumor epithelium. **c** Squamous metaplasia is observed in tumor cells. **d** The tumor invaded the skeletal muscle. **e** Acne-like necrosis was observed in the tumor cells. **f** Nerve invasion. **g** Gingival tissue (HE × 20). **h** Malignant transformation of ameloblastoma in the gingival tissue (HE × 100)

**Fig. 4:**
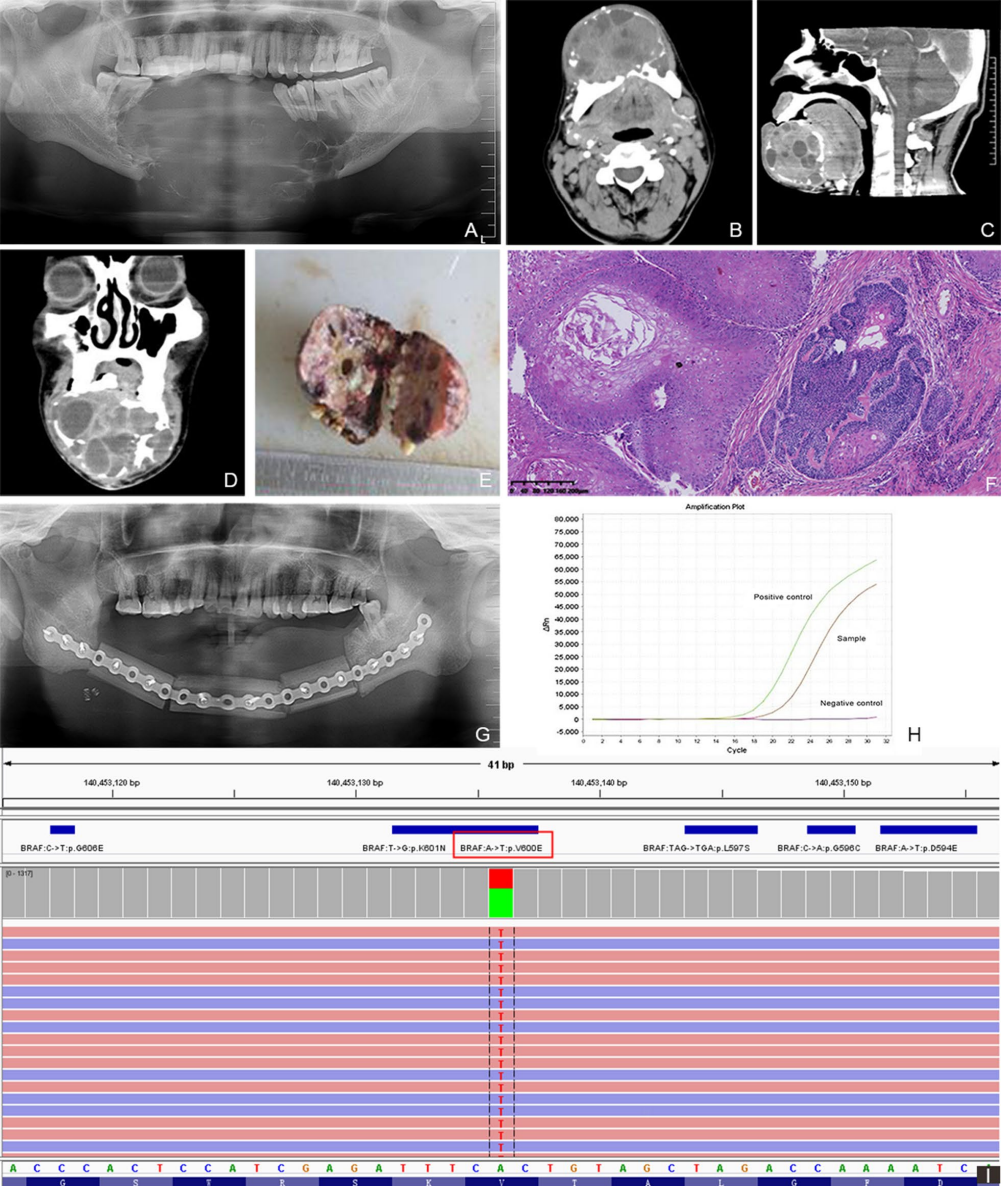
Patient 7, Primary ameloblastic carcinoma: **a** Radiography showed bone destruction in the mandible. **b**–**d** Computed tomography (CT) showed expansive destruction of the mandible in the axial, coronal, and sagittal view. Uneven soft tissue density, bone segregation, and uneven enhancement were observed. The lymph nodes in neck region I, II, and III on both sides were enlarged. **e** Postoperative samples showed a mixed cystic-solid structure and polycystic type. **f** Hematoxylin and eosin staining showed the carcinogenesis of the ameloblastoma with squamous cell carcinoma. **g** Transplantation of fibular myocutaneous flaps was simultaneously performed after radical tumor resection. **h**, **i** *BRAF*-*V600E* mutation

**Fig. 5:**
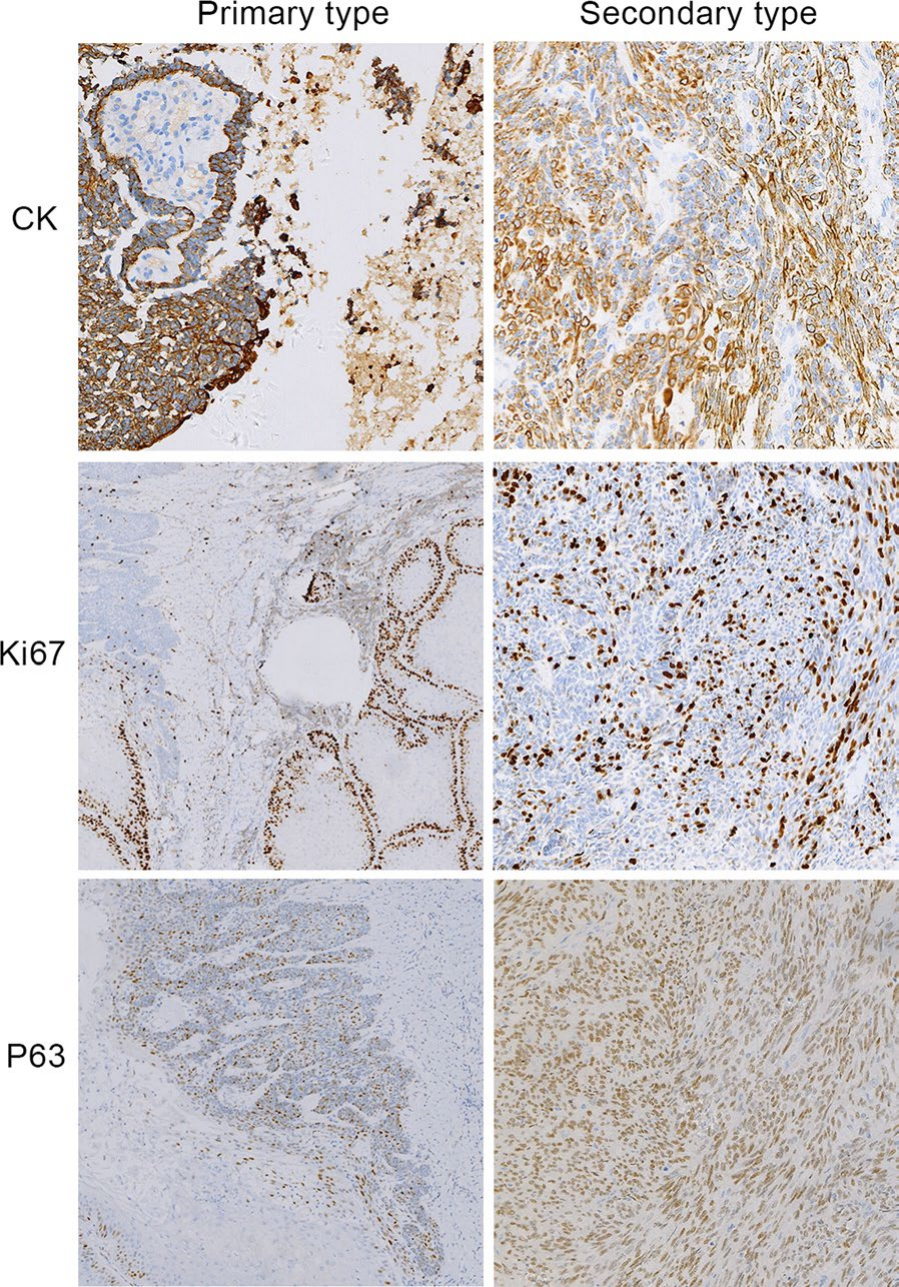
Immunohistochemical comparison of primary and secondary ameloblastic carcinomas

AC can retains some of the typical histologic features of benign ameloblastoma, but it mainly presents malignant features, such as atypia, local necrosis, and perineural infiltration (Fig. 3). Peripheral AC is a malignant transformation of primary ameloblastoma that occurs outside the bone in the early stage. The malignant histological features of ameloblastoma can be observed in gingival tissues (Fig. 3g, h). Primary AC has histological characteristics similar to benign ameloblastoma but with obvious local cell atypia (Fig. 4f). Immunohistochemistry revealed that the proliferation index of Ki-67 in secondary tumors was higher than that of Ki-67 in primary tumors (Fig. 5). Furthermore, *BRAF-V600E* was detected in all 5 patients that underwent testing. *BRAF* genetic testing was not performed in the remaining 10 patients due to DNA degradation in the tissue samples (Table 1).

## Discussion

AC is a rare and widely invasive malignant odontogenic epithelial neoplasm with significant proliferation and metastatic potential, requiring radical surgical intervention and close post-operative medical follow-up [9]. Little is known about the malignant mechanism of AC. A mixture of benign and malignant features may be present within the same tumor. Karakida et al. [4] inferred that postoperative chronic inflammation may promote its malignant transformation. Slater [10] proposed that multistep carcinogenesis, as seen in secondary AC, develops from pre-existing benign ameloblastoma before malignant transformation; patients usually experience multiple recurrences and various management courses. Accordingly, its diagnosis and treatment remain challenging. In this study, AC showed a unique biological behavior, different from ameloblastoma, which can not only cause extensive destruction of the jaw bone, but also nerve paralysis and distant metastasis. Imaging and histological features also showed that it had a more aggressive biological behavior.

A wide incidence age range with a mean age of 49 years has been reported [2]. In this study, the median presentation age was 53 years. The mandible was the most common AC location, closely correlating with earlier findings, which showed the posterior part of the mandible to be the most affected site, followed by the maxilla [11]. In this study, only two patients presented with cervical lymph nodes and lung metastases. Giridhar et al. [2] found that the progression-free survival and overall survival of AC were not different for patients with or without neck dissection, and prophylactic neck node dissection should be avoided. In this study, one patient suffered from eight recurrences. For this phenomenon, an important factor may be the maxillary location because of the abundant blood supply and its adjacent location to vital structures including the orbit, cranial base, and pterygomaxillary fossa, which are difficult to access by the surgeon and to obtain clear surgical margins [12]. The nuclear protein, Ki-67 antigen is a reliable marker reflecting cell proliferation, and Ki-67 is more specific for the proliferation of ameloblastoma and AC [13]. In this study, immunohistochemistry revealed that the proliferation index of Ki-67 in secondary tumors was higher than that in primary tumors, but radiography revealed that primary tumors were more destructive than secondary tumors, indicating that the increase in the Ki-67 index could not explain the invasiveness and bone destruction of those lesions but could help explain its ability to sustain growth and expansion [14]. Therefore, using the Ki-67 index increase to illustrate the destructive ability of AC remains a subjective measure [15]. In this study, one patient had AC accompanied by squamous cell carcinoma. This may be due to the malignant transformation of acanthomatous ameloblastoma, which exhibits extensive squamous metaplasia [16, 17]. Although AC shows squamous cell differentiation, it is not its main component; therefore, the possibility of AC must be first considered, rather than a primary oral squamous cell carcinoma [18].

The early treatment of ameloblastoma is crucial, and its malignant potential should be considered. The treatment of AC is usually extensive local excision. If the identification of benign or malignant ameloblastoma before surgery is difficult, frozen histological examination should be carried out at multiple tumor boundaries during surgery to discover malignant features in time [19]. Neck dissection should be considered only when local metastasis is suspected on clinical examination. In this study, radical resection and jaw reconstruction proved effective in reducing the recurrence and improving the quality of life of the patients. Radiotherapy is a classic adjuvant method for treating partially resected tumors; however, its efficacy is still unclear [8, 20–22], as is that of systemic chemotherapy. Currently, various chemotherapeutic drugs, including platinum cyclophosphamide, carboplatin, paclitaxel, and 5-fluorouracil, have been reported useful, although with unsatisfactory therapeutic effects [23, 24]. In a previous report, an 8-year-old child was diagnosed with AC and systemic metastases and died after 5 cycles of chemotherapy [24]. The recent development of molecular biotechnology has improved tumor treatment. The incidence of *BRAF*-*V600E* mutations is high in osteogenic tumors [5–7, 25]. *BRAF* mutation is also associated with ameloblastoma invasiveness [26], and our results also demonstrated that *BRAF-V600E* is associated with AC. Furthermore, Kaye et al. [25] once treated a patient with ameloblastoma and pulmonary metastases by using two targeted drugs, dabrafenib and trametinib, which inhibit the effects of *BRAF* mutation. After 20 weeks, both the primary oral and pulmonary metastases were responding to treatment, suggesting that *BRAF-V600E* may be a therapeutic target for ameloblastoma, and targeted drug therapy may be used for AC with *BRAF-V600E* mutations.

There are some limitations to our study. Due to the complex mechanism of malignant transformation in AC, more studies focused on AC samples in various fields, such as molecular pathology and molecular biology, should be performed. Due to its rarity, AC treatment with molecular-targeted drugs are still untested. Thus, more AC cases need be documented.

## Conclusion

AC diagnosis should be combined with clinical, imaging, and pathological manifestations to improve diagnostic accuracy. Due to its rarity, there is little knowledge about AC’s diagnosis and management. Moreover, clinical, imaging, and pathological features refer only to phenotypic characteristics. Further research on the mechanism of malignant transformation will help us to develop new treatment methods for this disease.

## Methods

This study was approved by the Medical Ethics Review Committee of the First Affiliated Hospital of Zhengzhou University (Approval No: KY-2019-LW-008).

Data of 15 patients with AC from the First Affiliated Hospital of Zhengzhou University from 2014 to 2019 were reviewed. The medical files of all patients from the first consultation to the last medical consultation were collected. Hematoxylin and eosin (H&E) staining was performed on 4 μm histological sections and reviewed by three pathologists with > 5 years of work experience to confirm the original diagnoses, following the 2017 WHO odontogenic tumor guidelines [27]. We recorded the patient age and sex, tumor diameter, primary tumor site, patient symptoms, presence and location of metastases, imaging and pathologic features, treatment applied, follow-up information, and time of the last medical consultation. All patients were histologically examined and confirmed to have AC. Five patients (1, 2, 4, 9 and 11) were tested for the BRAF-V600E mutation. The other patients′ tissue samples were stored for too long and DNA degraded, so they could not be tested.

### Immunohistochemical staining

Formalin-fixed, paraffin-embedded tissues from cases of AC were retrieved from the department of pathology, the first affiliated hospital of zhengzhou university. These tissues were cut into 4-μm tissue sections. Antibodies against the following antigens were used in this experiment: cytokeratin(CK) (mouse monoclonal antibody, AE1/AE3, Ready-to-use), P63 (mouse monoclonal antibody, 4A4 + UMAB4, Ready-to-use) from ZSGB-Bio, Beijing, China. Ki-67 (mouse monoclonal antibody, 30–9,Roche, Basel, Switzerland)is detected in Roche automatic immunohistochemistry platform.

### Real-time PCR analysis and DNA sequencing

Real-time PCR was performed using an ABI 7300 real-time PCR system (Applied Biosystems, Foster City, CA, USA) and the SYBR Premix Ex Taq reagent kit (Takara Bio, Inc., Shiga, Japan). The forward and reverse primers were 5′-TGCTTGCTCTGATAGGAAAATG-3′ and 5′-CCACAAAATGGATCCAGACA-3′, respectively. The reaction procedure was as follows: pre-denaturation at 95 °C for 3 min; denaturation at 94 °C for 30 s, annealing extension at 60 °C for 30 s, and amplification at 72 °C for 30 s, for a total of 35 cycles. The PCR reaction product was handed over to Wuhan Sevier Biotechnology Co.Ltd (Hubei, China) to complete the DNA sequencing process based on ABI 3730XL sequencer(Applied Biosy-stem Inc, Waltham, Massachusetts,US).

## Data Availability

The datasets used and/or analyzed during the current study are available from the corresponding author on reasonable request.

## Abbreviations

AC: Ameloblastic carcinoma
H&E: Hematoxylin and eosin
WHO: World Health Organization
